# Abscopal Effect after Palliative Radiation Therapy for Metastatic Adenocarcinoma of the Esophagus

**DOI:** 10.7759/cureus.3089

**Published:** 2018-08-02

**Authors:** Moss Bruton Joe, Pauline T Truong

**Affiliations:** 1 Radiation Oncology, Vancouver Island Center/BC Cancer Agency, Vancouver, CAN; 2 Radiation Oncology, University of British Columbia/BC Cancer Agency, Vancouver, CAN

**Keywords:** bystander/abscopal effect, esophageal cancer, radiation therapy, immunotherapy, lymph node metastasis

## Abstract

The abscopal effect is a rare phenomenon in the treatment of metastatic cancer where tumor regression occurs distant from the irradiated volume. It is thought that local radiation induces immunogenic cell death by systemically enhancing the host’s antitumor immune system. We present a rare case of the abscopal effect in esophageal adenocarcinoma. After palliative radiation therapy to the primary tumor and adjacent lymph nodes, a complete response was observed not only in the irradiated tissues, but also in non-irradiated metastatic lymph nodes. The patient remains cancer-free one year later. A better understanding of the abscopal effect may lead to novel research to improve patient outcome in the often dismal case of esophageal adenocarcinoma.

## Introduction

Worldwide, an estimated 456,000 new esophageal cancer cases and 400,000 deaths have been documented in 2012 [1]. Squamous cell carcinoma and adenocarcinoma make up over 90% of esophageal cancer cases. Unlike many other cancer types, esophageal adenocarcinoma (EA) has increased in incidence over the last few decades and is expected to continue this upward trend – an increase of 140% over the next 10 years, making it the fastest growing cancer in North America [2]. Survival outcomes for advanced stage EA are poor, creating the need for new treatment strategies.

The term “abscopal effect” was coined by Mole in 1953 to describe the systemic effects of radiation therapy on tissues distant from the irradiated volume [3]. We present a rare case of the abscopal effect in a patient with esophageal adenocarcinoma treated with palliative radiation therapy. Potential implications for research in immune response to cancer and immunotherapeutics that may lead to improved clinical outcome will be discussed.

## Case presentation

**Figure 1:**
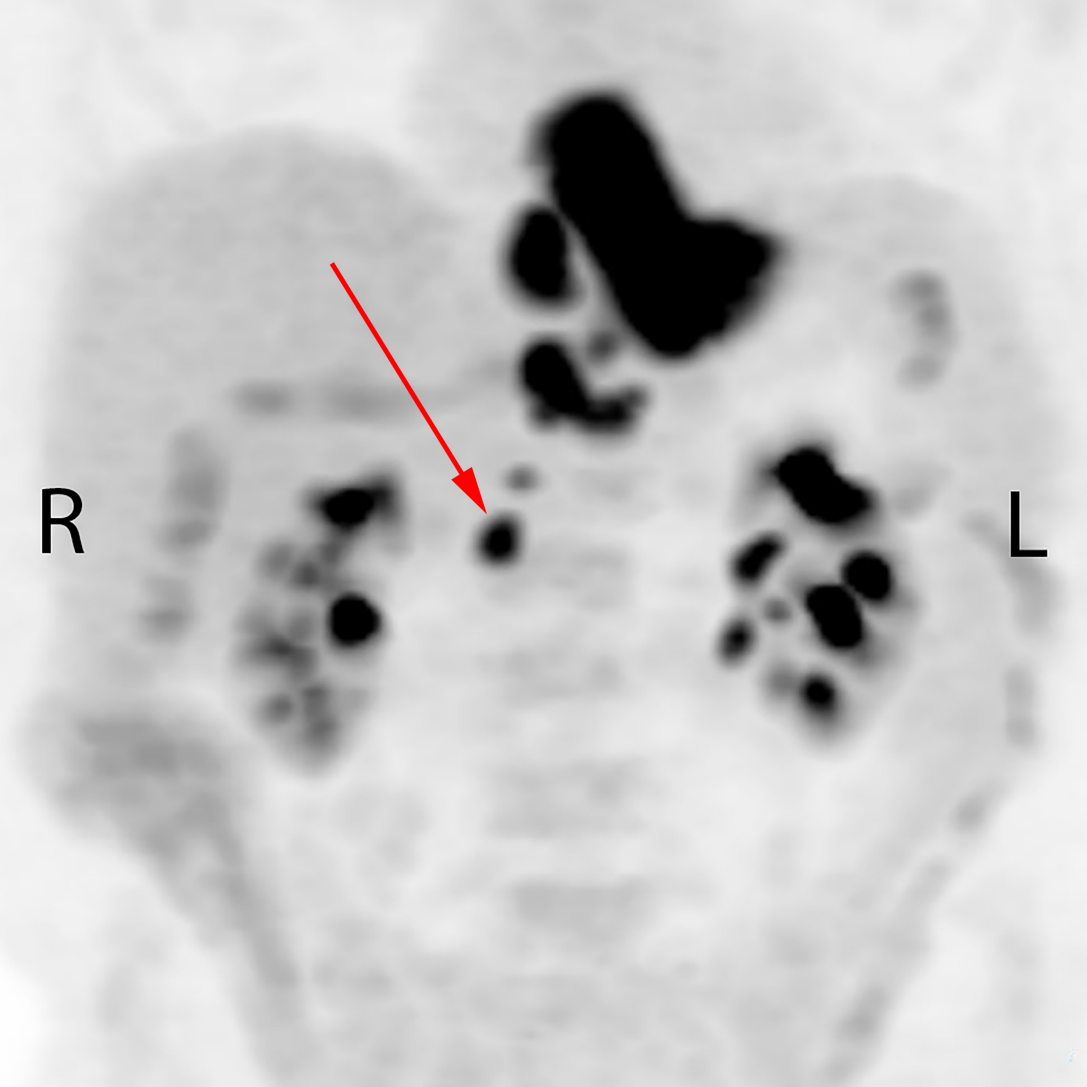
Pre-treatment staging positron emission tomography showing primary tumor and nodal disease involving the paraesophageal regions and upper abdomen extending to the level of the right renal vessels. The red arrow indicates the untreated node.

A 74-year-old male presented in February 2016 with increasing dysphagia. There was no history of prior abdominal infection or surgery. On esophagogastroscopy, a necrotic and circumferential friable tumor was seen at 33 to 40 cm from the incisors, with an endoscopic appearance of involvement of gastroesophageal (GE) junction and the proximal 2 cm of the stomach. Biopsies of the distal esophageal tumor confirmed poorly differentiated adenocarcinoma. The patient was anemic with a hemoglobin of 89 g/L. Staging endoscopic ultrasound suggested a breach of muscularis propria and four enlarged paraesophageal nodes. Neoadjuvant chemoradiotherapy followed by esophagectomy was initially considered; however, a staging positron emission tomography (PET) scan demonstrated 18-fluorodeoxyglocose (FDG) uptake not only in the primary tumor, but also in the paraesophageal region near the GE junction and upper abdominal lymph nodes extending as far inferiorly as the right renal vessels, in a retrocaval location (Figure 1).

A radiation oncology consultation was sought regarding treatment options of such extensive lymphadenopathy. Palliative radiation therapy (RT) was recommended. The patient was also evaluated by a medical oncologist who advised that chemotherapy may be considered after assessing the response to palliative radiotherapy.

**Figure 2:**
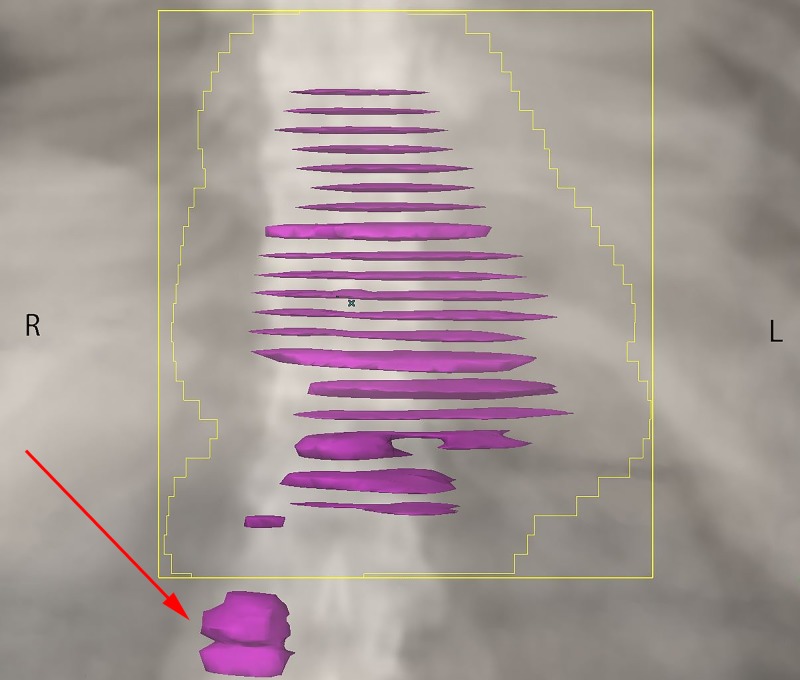
Palliative radiation treatment volume, targeting the primary tumor and paraesophageal nodes only. The red arrow indicates the untreated node.

From March 21, 2016 to April 5, 2016, the patient received palliative RT to the symptomatic primary tumor and closest adjacent nodes using a pair of anterior and posterior fields. A total dose of 30 Gray (Gy) was prescribed over 10 daily fractions. As the lymphadenopathy in the lower abdomen was not symptomatic, and would have contributed to increased toxicity, this region was deliberately excluded from the high dose RT volume (Figure 2). Other than very mild odynophagia, the patient had no other RT-related side effects. On the first follow-up visit, one month following treatment completion, he had improved swallowing function and a weight gain of six pounds.

Follow-up computed tomography (CT) scan was obtained on May 24, 2016 to evaluate for the suitability of chemotherapy and to serve as a baseline during systemic therapy. This demonstrated persistent thickening of the lower esophagus, with lymphadenopathy reported to have decreased in size and no significant retroperitoneal adenopathy. When given the option of receiving palliative chemotherapy, the patient declined and chose to continue on observation only. Further CT scans in August and October 2016 showed a complete response in the irradiated primary tumor and nodes, with a stable 10 mm lymph node at the right renal vein.

In January 2017, due to symptoms of increasing dysphagia, the patient was assessed by a thoracic surgeon for consideration of esophageal stent placement. Endoscopy on January 12, 2017 noted that there was a possible small amount of residual tumor at the GE junction, but there was no significant narrowing or stricture, and no biopsies were taken. A further CT scan on April 10, 2017 showed minor circumferential thickening of the distal esophagus, but unchanged from previous. Paraesophageal lymphadenopathy was reported to be unchanged. The PET-positive lymph node at the renal vein decreased from 10 mm to 5 mm.

**Figure 3:**
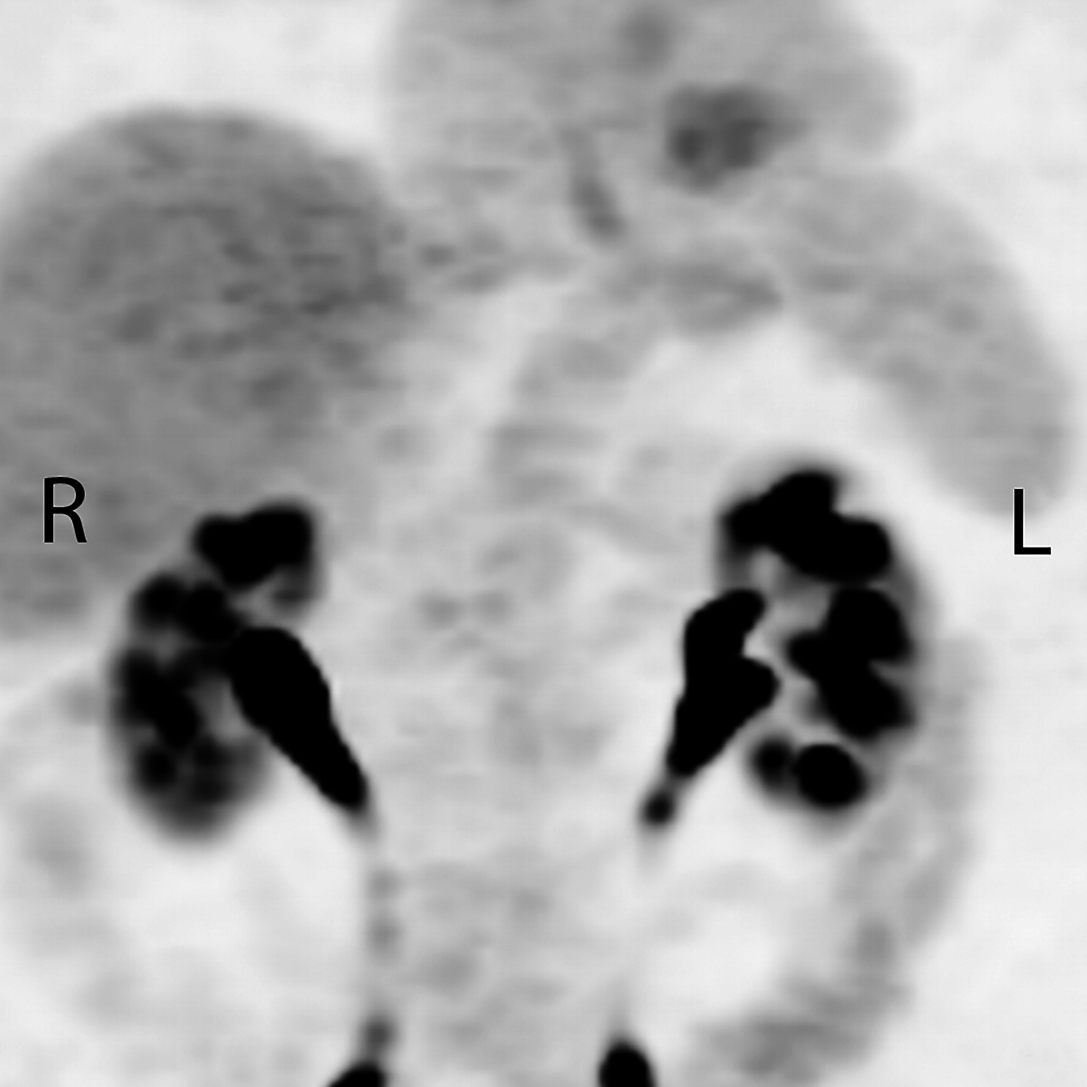
Post-treatment positron emission tomography showing resolution of all primary tumor and nodal disease, including nodes outside the radiation treatment volume.

The patient’s symptom of dysphagia resolved spontaneously, and an evaluation was made with a further PET scan on May 19, 2017 (Figure 3). This demonstrated mild residual FDG activity within the distal esophagus, more likely inflammatory change rather than malignancy. The FDG activity within all the lymph nodes, both treated and untreated, had unexpectedly resolved.

## Discussion

The abscopal, or bystander, effect is a rare outcome in metastatic cancer treatment. It is the response of a tumor outside of a recently irradiated area [3]. The abscopal effect has been reported in approximately 60 cases, primarily in lymphomas, melanomas and renal cell carcinomas [4]. There is only one other documented case in EA. There, palliative radiotherapy was administered and regression of lung metastases was sustained for 20 months [5].

Treatment for EA depends on tumor location, disease stage, patient preferences and comorbid conditions. Over 90% of cases are diagnosed in advanced stages, rendering treatment options limited. For patients with incurable metastatic disease, the modality of palliative treatment depends upon the location of disease, symptoms, tumor characteristics as well as the ability of the patient to tolerate side effects of therapy [6]. The five-year progression-free survival in patients with metastatic disease is poor, approximately 3% [7].

In the current case, tumor regression in lymph nodes both within and distant from the RT volume suggests that this is a case of the abscopal effect. The mechanism behind the abscopal phenomenon remains unclear, but a variety of hypotheses have been suggested. It is generally thought that the radiation interacts with the primary tumor site and its microenvironment to revert some immune-suppressive characteristics, allowing the immune system to better recognize tumors at distant sites [8, 9]. It may be that the irradiated tumor acts as a vaccine, causing anti-tumor immune cells to act systemically [8]. There is still debate in the field on which immune cells are responsible. Suggestions include improved antigen presentation and expression, enhanced function and movement of T-cells, upregulated check-point inhibition, and altered cytokine expression [8, 9].

The need for improved treatment for patients with metastatic esophageal cancer and other disease sites has led to a number of ongoing trials in immunotherapy, where the body’s pre-existing immune mechanisms are harnessed to target cancer cells. Researchers are optimistic because this treatment has the potential for longer lasting effects and minimized off-target harm [10]. The role of the immune system in the abscopal effect further supports research in this avenue.

Studies reporting preliminary success of immunotherapeutics in esophageal cancer and other tumor sites have emerged [9, 10]. Antibodies have been shown to successfully target immune checkpoints in melanoma and lung cancers, both of which have similarly high somatic mutations to EA. For example, a single patient demonstrated a long-term response in a trial using tremelimumab, a human antibody, against Cytotoxic T-lymphocyte-associated molecule-4, an immune checkpoint. Other studies have used antibodies to mark cancer cells for targeting by immune effector cells. One trial successfully used trastuzumab, a monoclonal antibody, to identify human epidermal growth factor receptor 2, an antigen overly expressed in some EAs [10]. Vaccine-based trials have shown upregulation of circulating antitumor CD8+ T cells after injection with tumor-associated proteins and peptides [9]. Another emerging method is adoptive cell therapy in which tumor-specific T cells are removed from the patient, amplified, and re-administered [10]. This has been successful in other malignancies such as renal cell carcinoma, lung cancer, melanoma, ovarian cancer, and glioma [9, 10].

Our case report highlights the synergistic interaction between radiation and immunogenic cell death. While immunotherapy was not applicable in this case as the patient declined systemic therapy, future studies of radiation in combination with immunotherapy warrant evaluation as a strategy that may improve EA outcomes. Because the success of immunotherapeutics requires a competent immune system, more research is needed to identify suitable candidates. Another area for future research could examine the optimization of dose and timing of radiation in relation to immunotherapy.

## Conclusions

We have presented a rare case of the abscopal effect in esophageal adenocarcinoma. Here, a palliative dose of radiation to the primary tumor and adjacent nodes resulted in a durable complete response of cancer in the irradiated field and non-irradiated metastases. The recent success in immunotherapy trials along with abscopal reports makes combined radiation and immunotherapy a promising area of research. Understanding the abscopal effect and immune mechanisms associated with EA may lead to innovative research to improve esophageal cancer outcomes.
